# The Uses of Rubber

**Published:** 1862-04

**Authors:** 


### THE USES OF RUBBER.

THE article in the present number of the Register, on the uses of rubber, by Dr. Richardson, we commend to the careful consideration of our readers. We think this is only a beginning in this direction. There are perhaps a great many uses to which rubber may be applied, that have not yet occurred to any one. We hope that any one to whom any thing new in this line occurs will follow the example set by Dr. R., will make it known at once, and by this means stimulate healthy emulation.

A new idea put forth by one begets new ideas in others.

